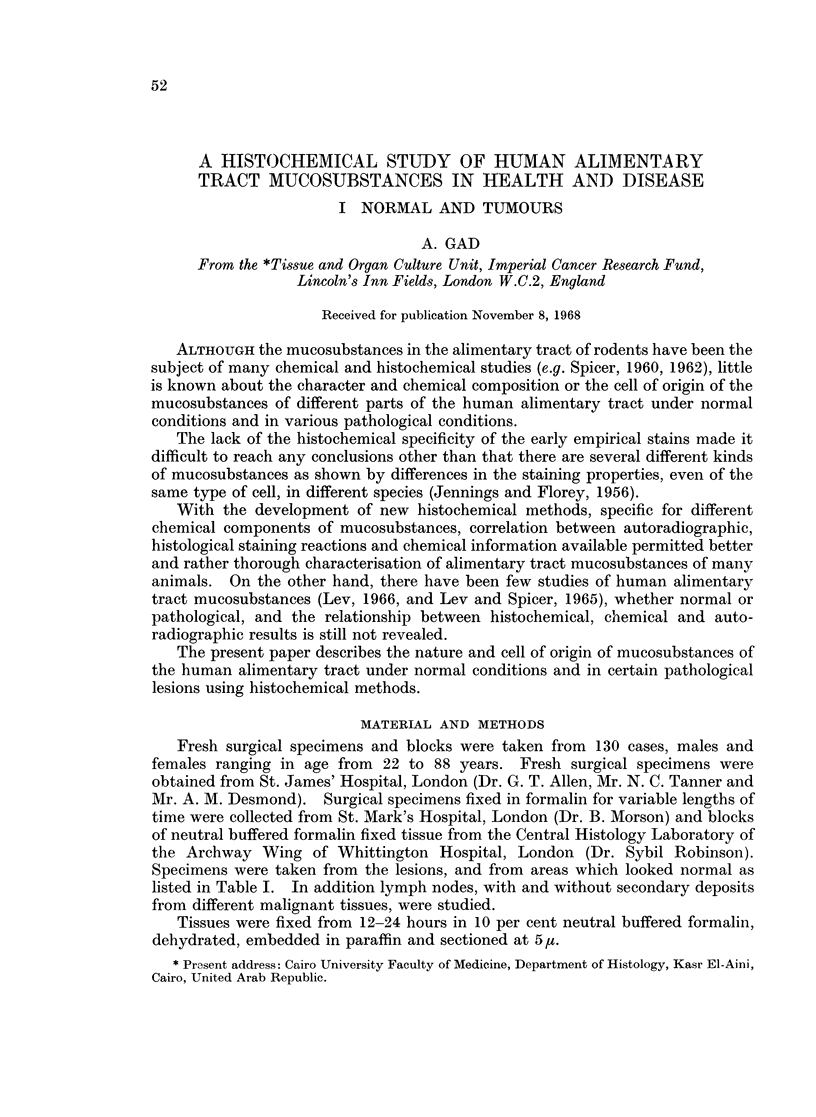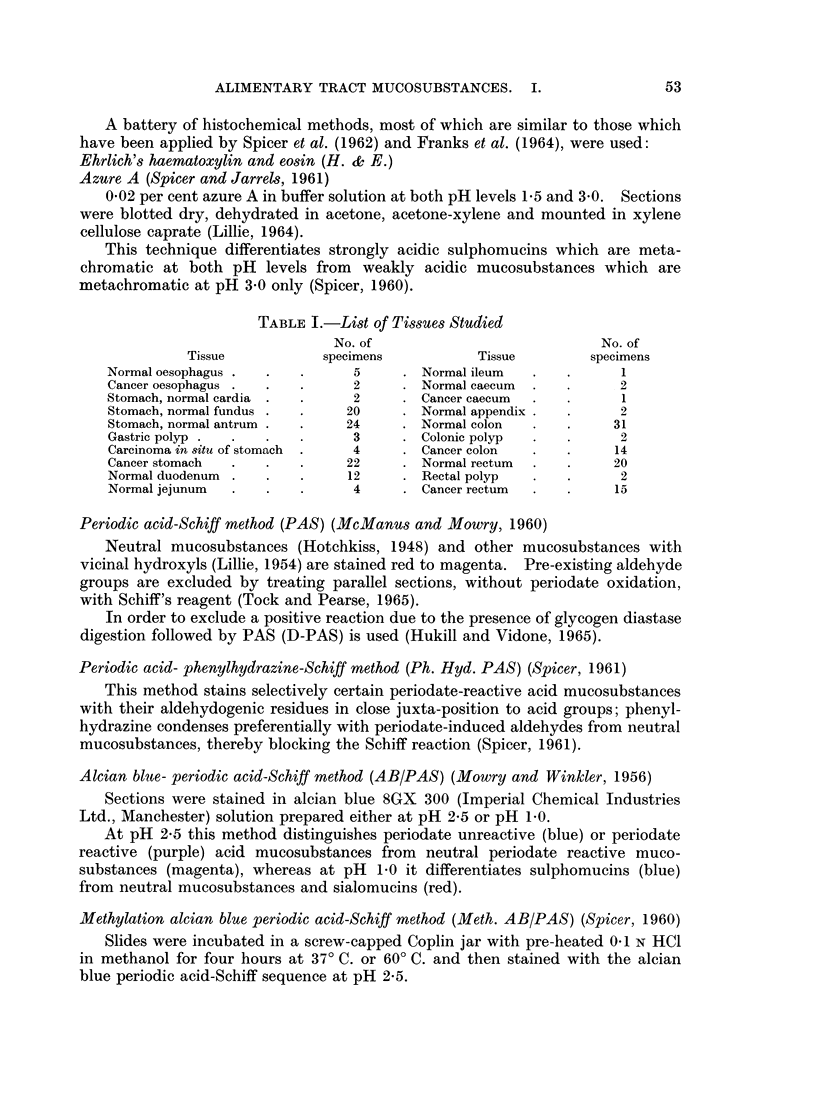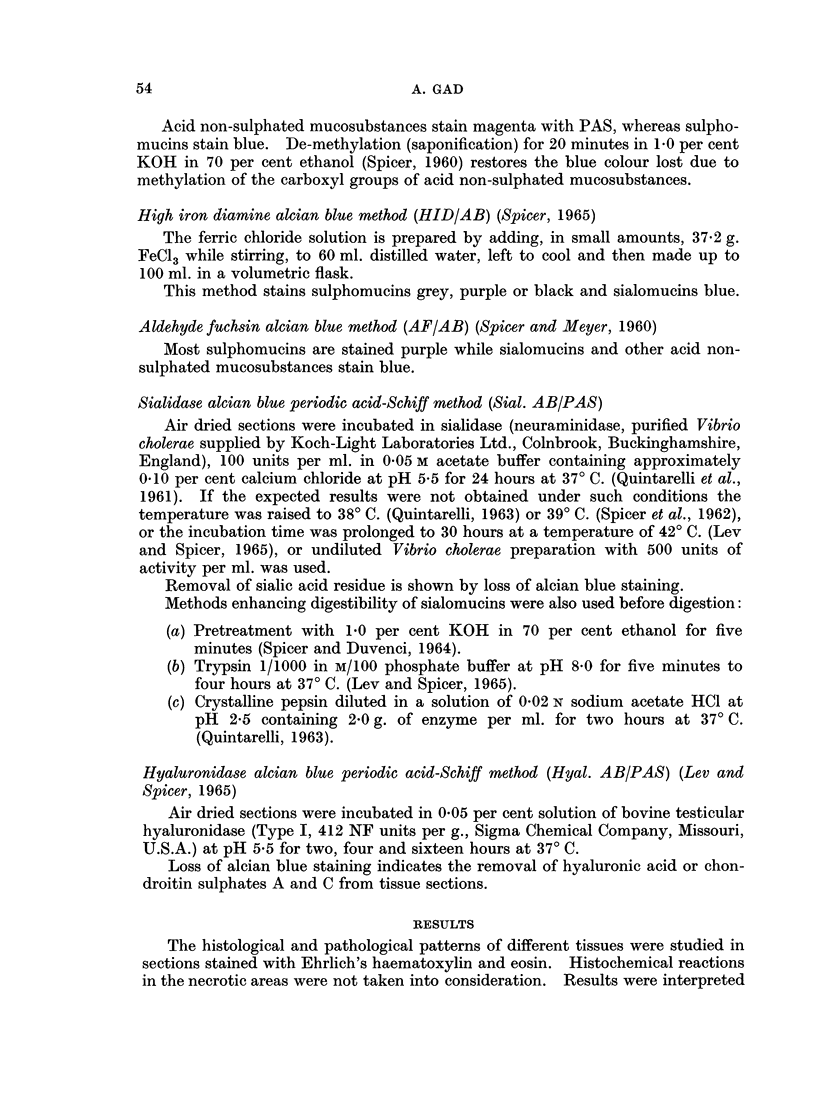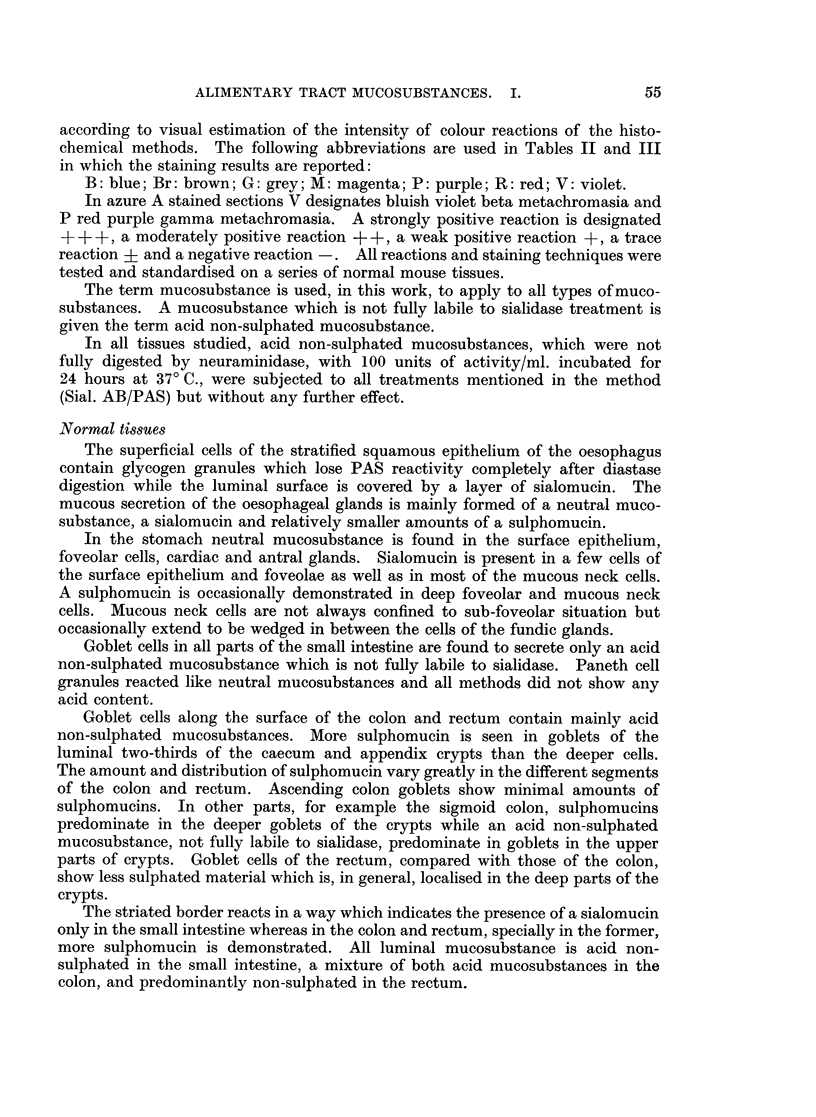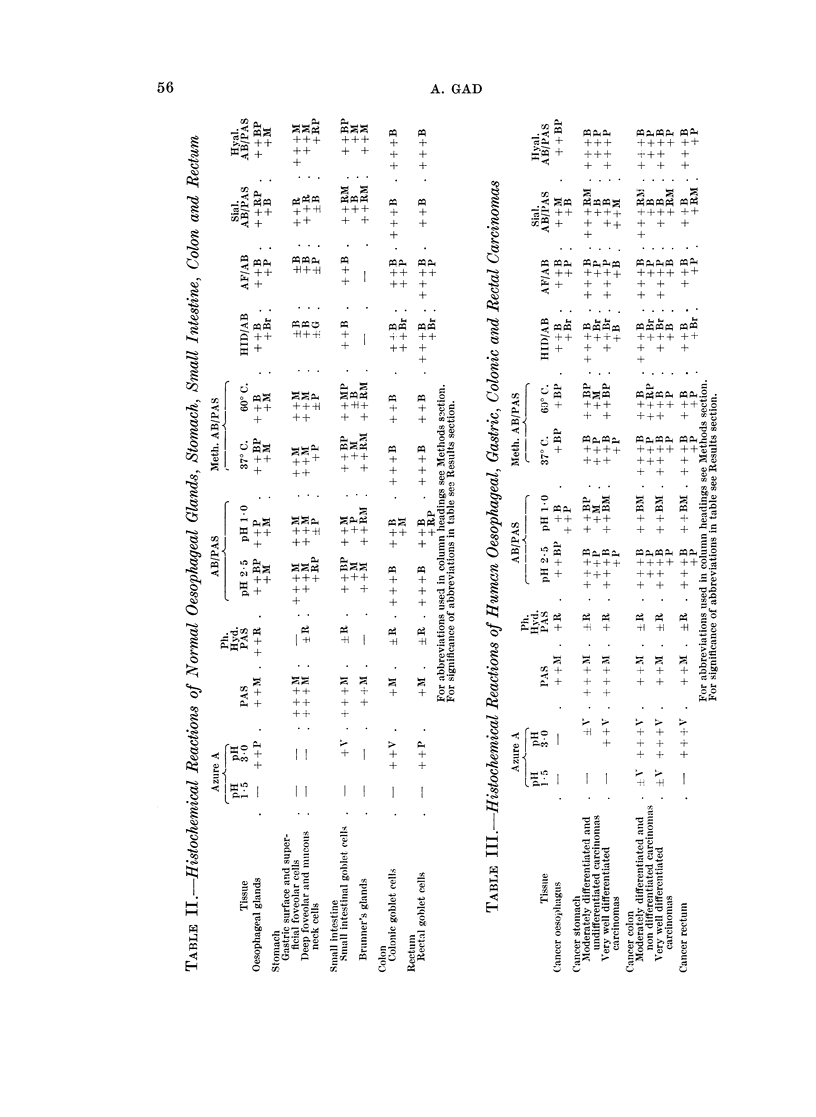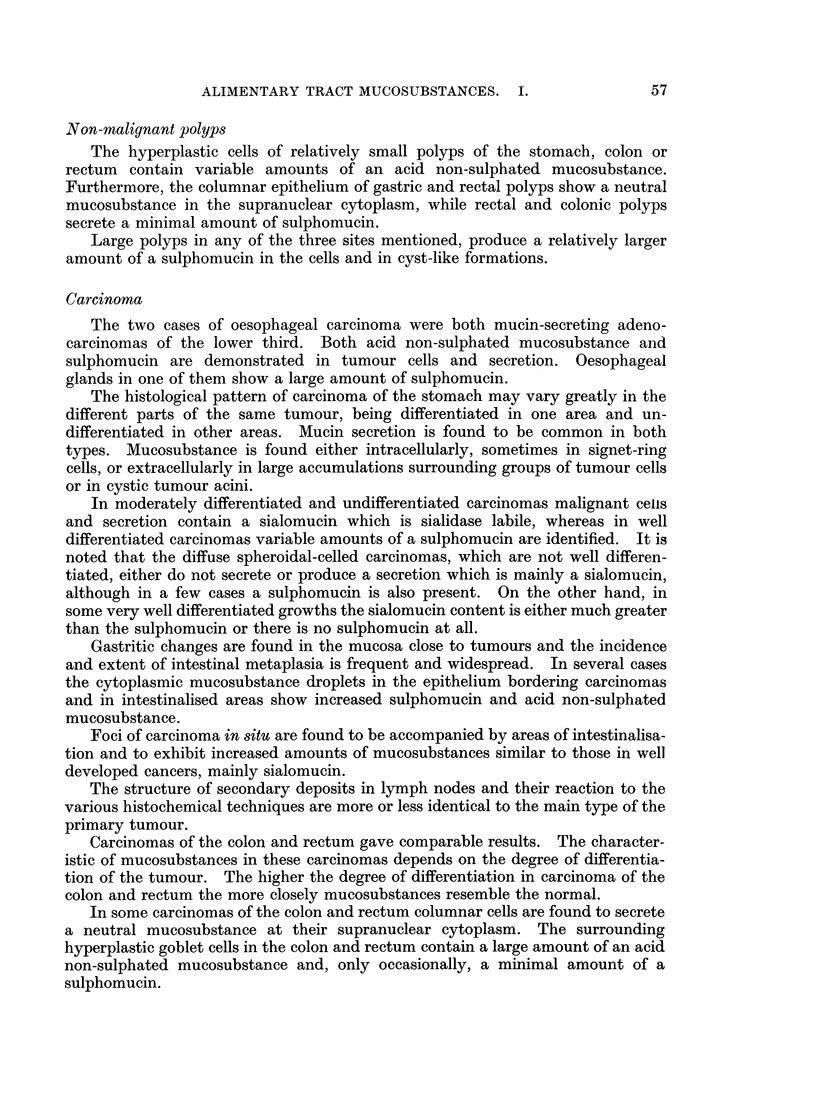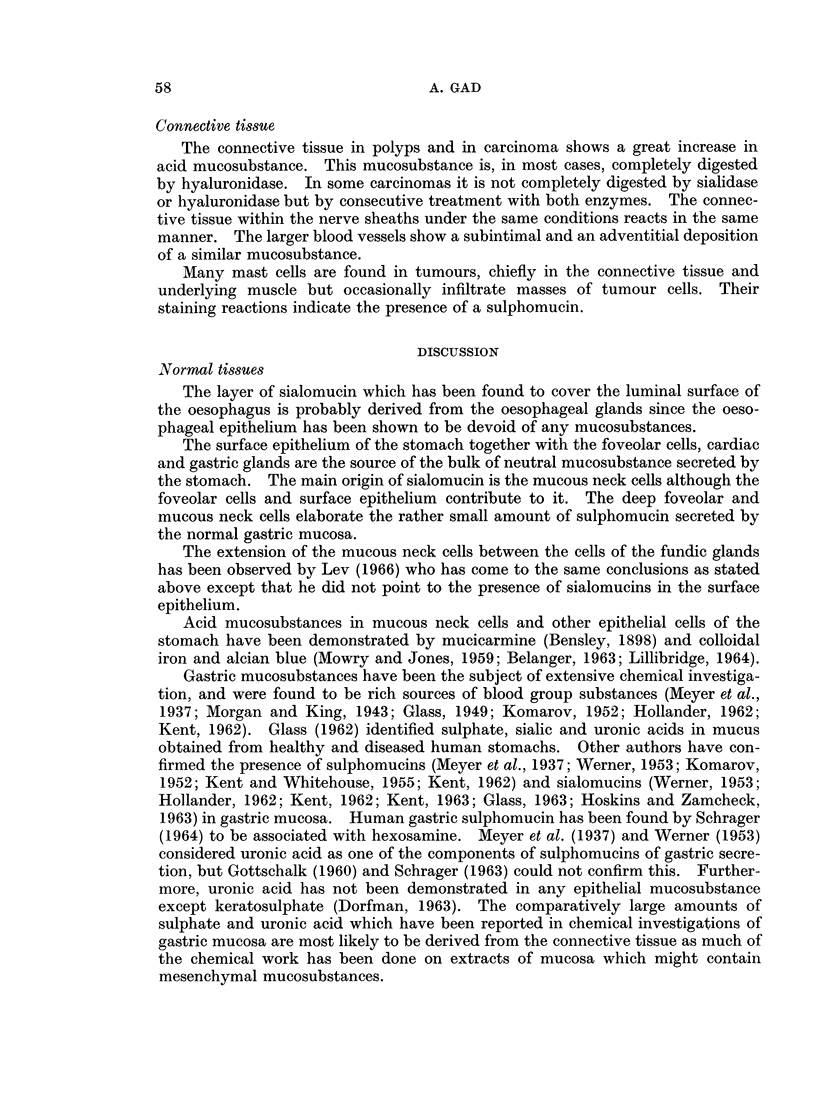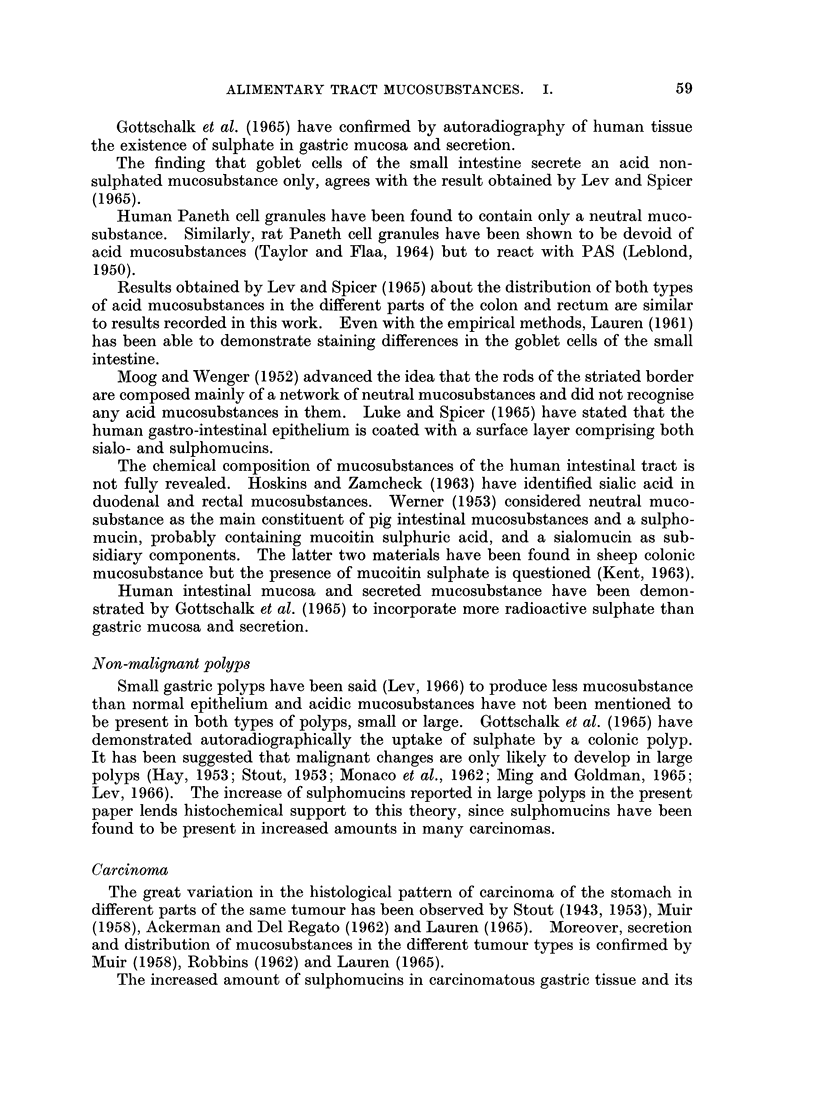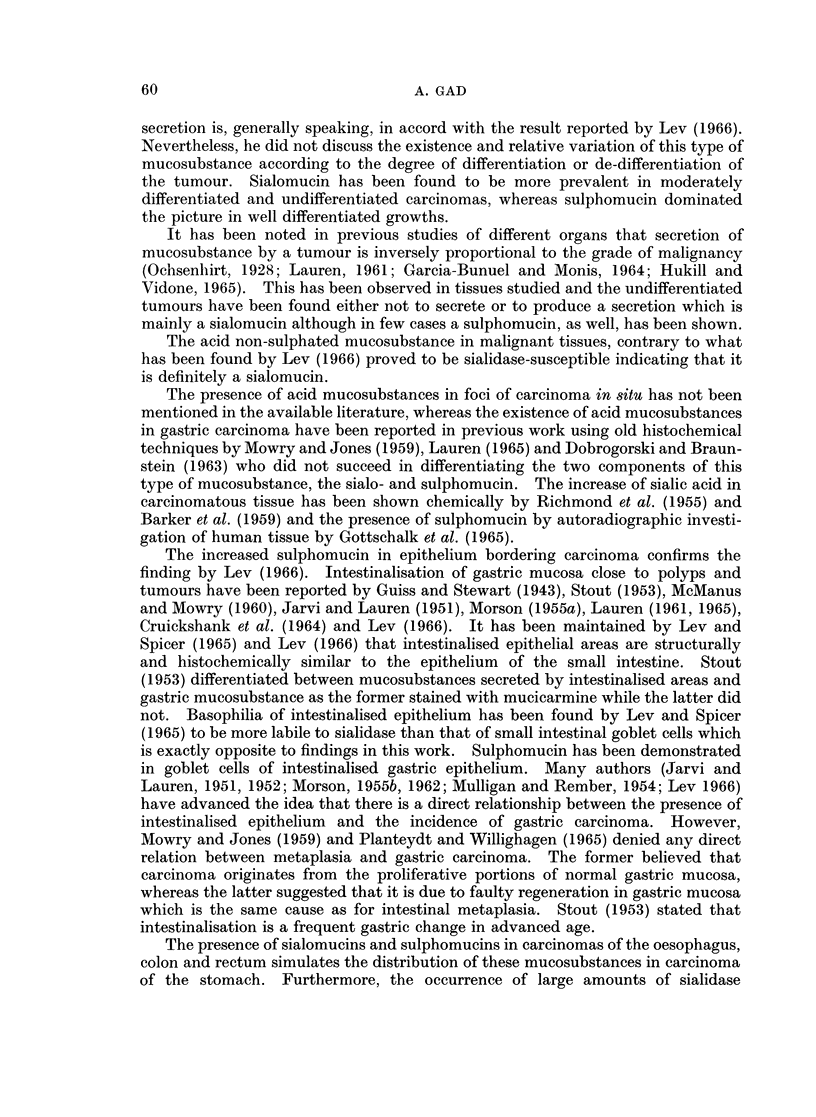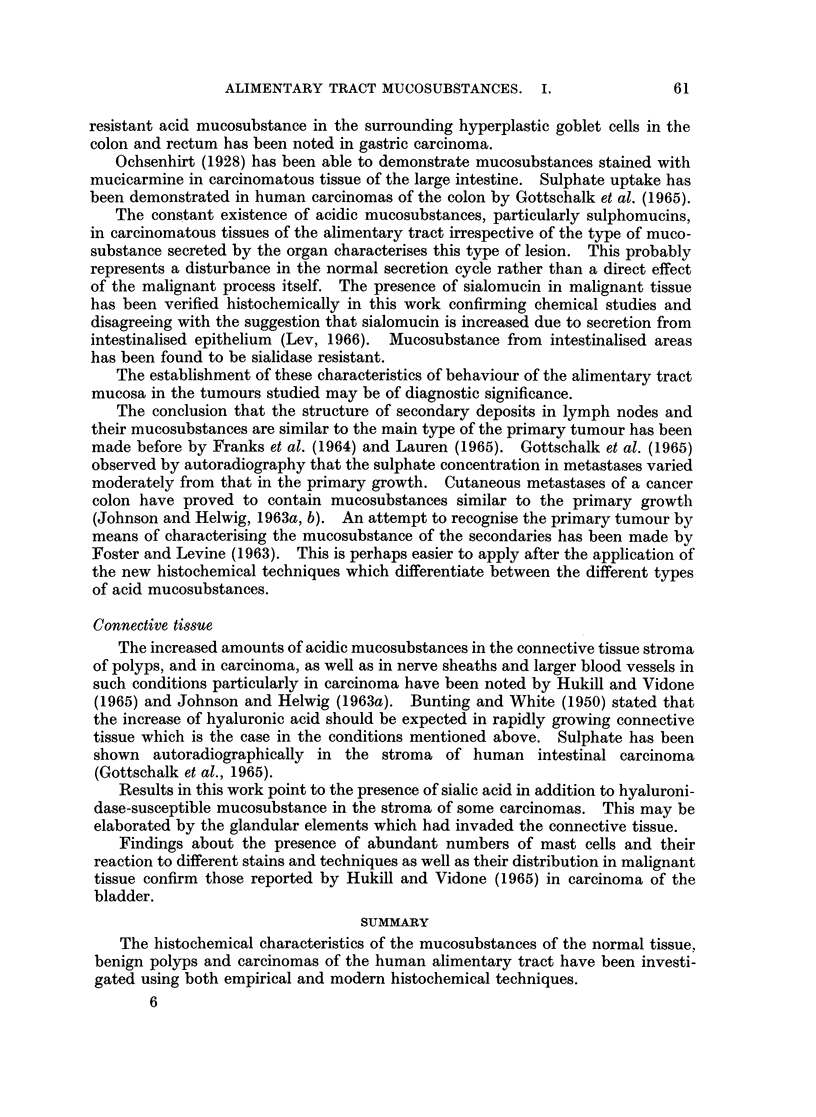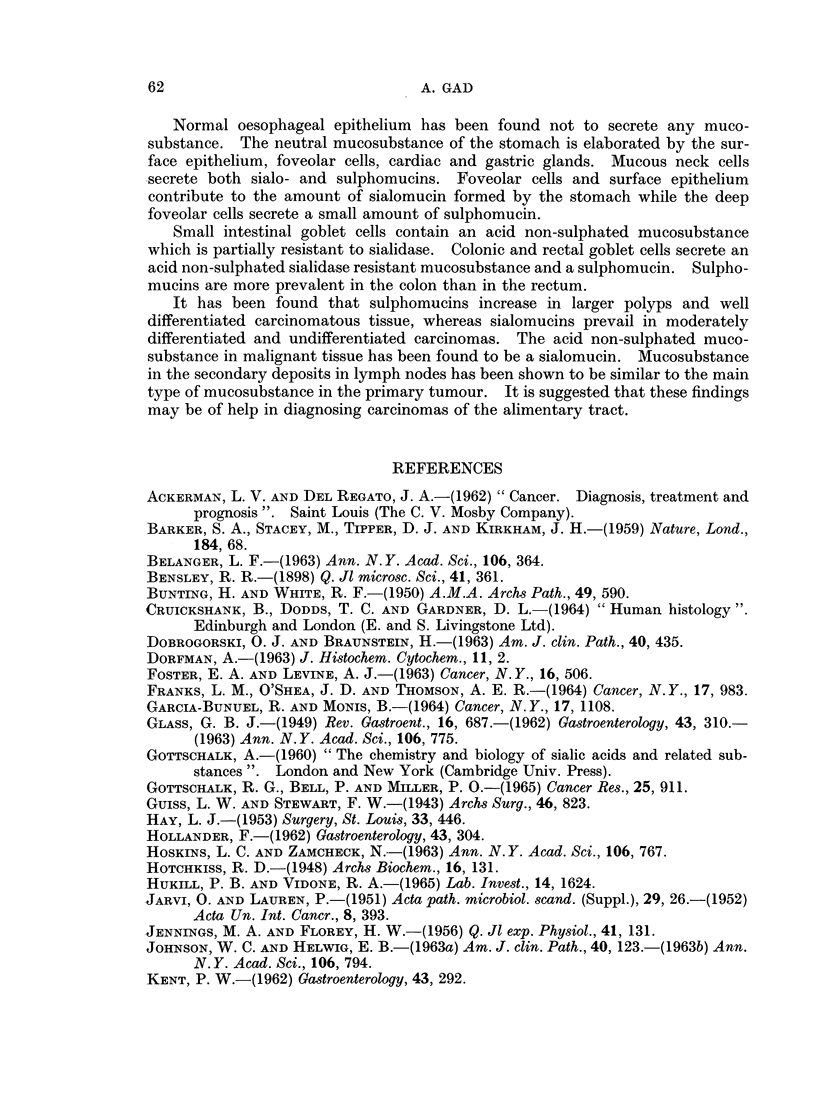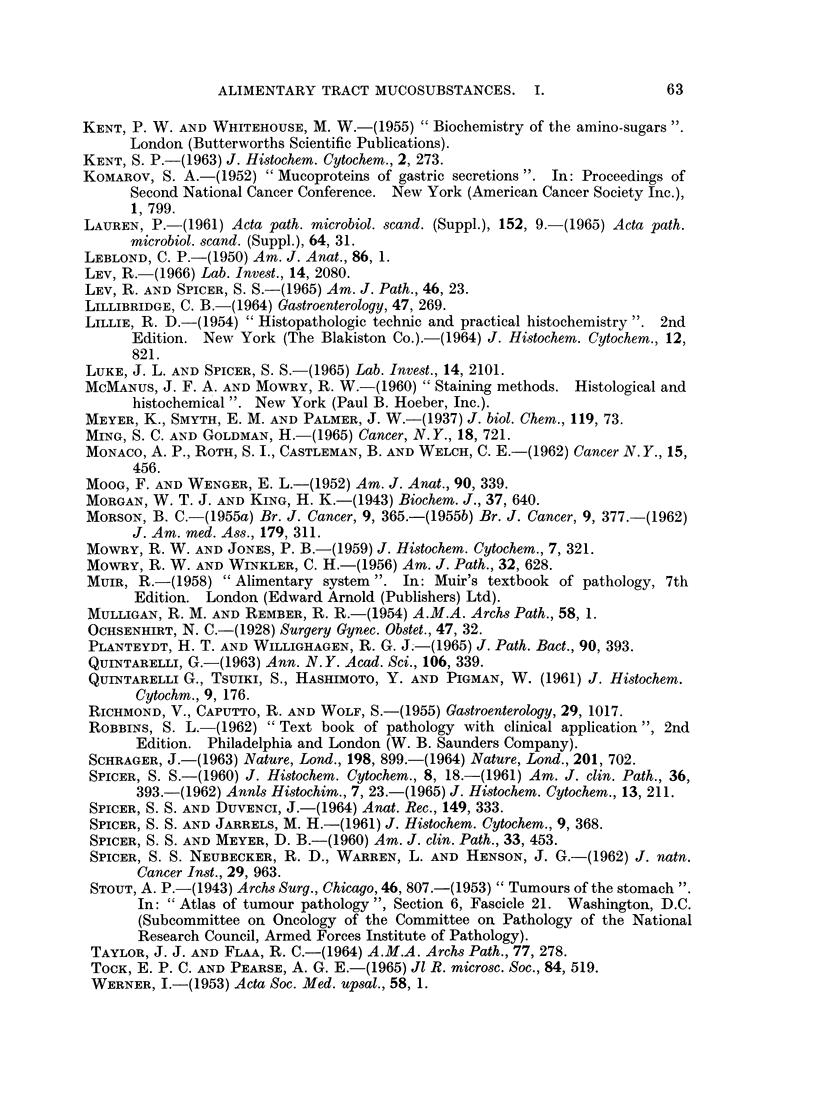# A histochemical study of human alimentary tract mucosubstances in health and disease. I. Normal and tumours.

**DOI:** 10.1038/bjc.1969.9

**Published:** 1969-03

**Authors:** A. Gad


### A HISTOCHEMICAL STUDY OF HUMAN ALIMENTARY TRACT MUCOSUBSTANCES IN HEALTH AND DISEASE

### I NORMAL AND TUMOURS

A. GAD

*From the *Tissue and Organ Culture Unit, Imperial Cancer Research Fund, Lincoln's Inn Fields, London W.C.2, England*

Received for publication November 8, 1968

ALTHOUGH the mucosubstances in the alimentary tract of rodents have been the subject of many chemical and histochemical studies (*e.g.* Spicer, 1960, 1962), little is known about the character and chemical composition or the cell of origin of the mucosubstances of different parts of the human alimentary tract under normal conditions and in various pathological conditions.

The lack of the histochemical specificity of the early empirical stains made it difficult to reach any conclusions other than that there are several different kinds of mucosubstances as shown by differences in the staining properties, even of the same type of cell, in different species (Jennings and Florey, 1956).

With the development of new histochemical methods, specific for different chemical components of mucosubstances, correlation between autoradiographic, histological staining reactions and chemical information available permitted better and rather thorough characterisation of alimentary tract mucosubstances of many animals. On the other hand, there have been few studies of human alimentary tract mucosubstances (Lev, 1966, and Lev and Spicer, 1965), whether normal or pathological, and the relationship between histochemical, chemical and autoradiographic results is still not revealed.

The present paper describes the nature and cell of origin of mucosubstances of the human alimentary tract under normal conditions and in certain pathological lesions using histochemical methods.

### MATERIAL AND METHODS

Fresh surgical specimens and blocks were taken from 130 cases, males and females ranging in age from 22 to 88 years. Fresh surgical specimens were obtained from St. James' Hospital, London (Dr. G. T. Allen, Mr. N. C. Tanner and Mr. A. M. Desmond). Surgical specimens fixed in formalin for variable lengths of time were collected from St. Mark's Hospital, London (Dr. B. Morson) and blocks of neutral buffered formalin fixed tissue from the Central Histology Laboratory of the Archway Wing of Whittington Hospital, London (Dr. Sybil Robinson). Specimens were taken from the lesions, and from areas which looked normal as listed in Table I. In addition lymph nodes, with and without secondary deposits from different malignant tissues, were studied.

Tissues were fixed from 12–24 hours in 10 per cent neutral buffered formalin, dehydrated, embedded in paraffin and sectioned at 5 $\mu$.

* Present address: Cairo University Faculty of Medicine, Department of Histology, Kasr El-Aini, Cairo, United Arab Republic.

A battery of histochemical methods, most of which are similar to those which have been applied by Spicer *et al.* (1962) and Franks *et al.* (1964), were used:

*Ehrlich's haematoxylin and eosin (H. & E.)*

*Azure A (Spicer and Jarrels,* 1961)

0·02 per cent azure A in buffer solution at both pH levels 1·5 and 3·0. Sections were blotted dry, dehydrated in acetone, acetone-xylene and mounted in xylene cellulose caprate (Lillie, 1964).

This technique differentiates strongly acidic sulphomucins which are metachromatic at both pH levels from weakly acidic mucosubstances which are metachromatic at pH 3·0 only (Spicer, 1960).

TABLE I.—*List of Tissues Studied*

| Tissue | No. of specimens | Tissue | No. of specimens |
|---|---|---|---|
| Normal oesophagus | 5 | Normal ileum | 1 |
| Cancer oesophagus | 2 | Normal caecum | 2 |
| Stomach, normal cardia | 2 | Cancer caecum | 1 |
| Stomach, normal fundus | 20 | Normal appendix | 2 |
| Stomach, normal antrum | 24 | Normal colon | 31 |
| Gastric polyp | 3 | Colonic polyp | 2 |
| Carcinoma *in situ* of stomach | 4 | Cancer colon | 14 |
| Cancer stomach | 22 | Normal rectum | 20 |
| Normal duodenum | 12 | Rectal polyp | 2 |
| Normal jejunum | 4 | Cancer rectum | 15 |

*Periodic acid-Schiff method (PAS) (McManus and Mowry,* 1960)

Neutral mucosubstances (Hotchkiss, 1948) and other mucosubstances with vicinal hydroxyls (Lillie, 1954) are stained red to magenta. Pre-existing aldehyde groups are excluded by treating parallel sections, without periodate oxidation, with Schiff's reagent (Tock and Pearse, 1965).

In order to exclude a positive reaction due to the presence of glycogen diastase digestion followed by PAS (D-PAS) is used (Hukill and Vidone, 1965).

*Periodic acid- phenylhydrazine-Schiff method (Ph. Hyd. PAS) (Spicer,* 1961)

This method stains selectively certain periodate-reactive acid mucosubstances with their aldehydogenic residues in close juxta-position to acid groups; phenylhydrazine condenses preferentially with periodate-induced aldehydes from neutral mucosubstances, thereby blocking the Schiff reaction (Spicer, 1961).

*Alcian blue- periodic acid-Schiff method (AB/PAS) (Mowry and Winkler,* 1956)

Sections were stained in alcian blue 8GX 300 (Imperial Chemical Industries Ltd., Manchester) solution prepared either at pH 2·5 or pH 1·0.

At pH 2·5 this method distinguishes periodate unreactive (blue) or periodate reactive (purple) acid mucosubstances from neutral periodate reactive mucosubstances (magenta), whereas at pH 1·0 it differentiates sulphomucins (blue) from neutral mucosubstances and sialomucins (red).

*Methylation alcian blue periodic acid-Schiff method (Meth. AB/PAS) (Spicer,* 1960)

Slides were incubated in a screw-capped Coplin jar with pre-heated 0·1 N HCl in methanol for four hours at 37° C. or 60° C. and then stained with the alcian blue periodic acid-Schiff sequence at pH 2·5.

Acid non-sulphated mucosubstances stain magenta with PAS, whereas sulphomucins stain blue. De-methylation (saponification) for 20 minutes in 1·0 per cent KOH in 70 per cent ethanol (Spicer, 1960) restores the blue colour lost due to methylation of the carboxyl groups of acid non-sulphated mucosubstances.

*High iron diamine alcian blue method (HID/AB) (Spicer, 1965)*

The ferric chloride solution is prepared by adding, in small amounts, 37·2 g. $FeCl_3$ while stirring, to 60 ml. distilled water, left to cool and then made up to 100 ml. in a volumetric flask.

This method stains sulphomucins grey, purple or black and sialomucins blue.

*Aldehyde fuchsin alcian blue method (AF/AB) (Spicer and Meyer, 1960)*

Most sulphomucins are stained purple while sialomucins and other acid non-sulphated mucosubstances stain blue.

*Sialidase alcian blue periodic acid-Schiff method (Sial. AB/PAS)*

Air dried sections were incubated in sialidase (neuraminidase, purified *Vibrio cholerae* supplied by Koch-Light Laboratories Ltd., Colnbrook, Buckinghamshire, England), 100 units per ml. in 0·05 M acetate buffer containing approximately 0·10 per cent calcium chloride at pH 5·5 for 24 hours at 37° C. (Quintarelli *et al.*, 1961). If the expected results were not obtained under such conditions the temperature was raised to 38° C. (Quintarelli, 1963) or 39° C. (Spicer *et al.*, 1962), or the incubation time was prolonged to 30 hours at a temperature of 42° C. (Lev and Spicer, 1965), or undiluted *Vibrio cholerae* preparation with 500 units of activity per ml. was used.

Removal of sialic acid residue is shown by loss of alcian blue staining.

Methods enhancing digestibility of sialomucins were also used before digestion:

(*a*) Pretreatment with 1·0 per cent KOH in 70 per cent ethanol for five minutes (Spicer and Duvenci, 1964).
(*b*) Trypsin 1/1000 in M/100 phosphate buffer at pH 8·0 for five minutes to four hours at 37° C. (Lev and Spicer, 1965).
(*c*) Crystalline pepsin diluted in a solution of 0·02 N sodium acetate HCl at pH 2·5 containing 2·0 g. of enzyme per ml. for two hours at 37° C. (Quintarelli, 1963).

*Hyaluronidase alcian blue periodic acid-Schiff method (Hyal. AB/PAS) (Lev and Spicer, 1965)*

Air dried sections were incubated in 0·05 per cent solution of bovine testicular hyaluronidase (Type I, 412 NF units per g., Sigma Chemical Company, Missouri, U.S.A.) at pH 5·5 for two, four and sixteen hours at 37° C.

Loss of alcian blue staining indicates the removal of hyaluronic acid or chondroitin sulphates A and C from tissue sections.

### RESULTS

The histological and pathological patterns of different tissues were studied in sections stained with Ehrlich's haematoxylin and eosin. Histochemical reactions in the necrotic areas were not taken into consideration. Results were interpreted according to visual estimation of the intensity of colour reactions of the histochemical methods. The following abbreviations are used in Tables II and III in which the staining results are reported:

B: blue; Br: brown; G: grey; M: magenta; P: purple; R: red; V: violet.

In azure A stained sections V designates bluish violet beta metachromasia and P red purple gamma metachromasia. A strongly positive reaction is designated $+++$, a moderately positive reaction $++$, a weak positive reaction $+$, a trace reaction $\pm$ and a negative reaction —. All reactions and staining techniques were tested and standardised on a series of normal mouse tissues.

The term mucosubstance is used, in this work, to apply to all types of mucosubstances. A mucosubstance which is not fully labile to sialidase treatment is given the term acid non-sulphated mucosubstance.

In all tissues studied, acid non-sulphated mucosubstances, which were not fully digested by neuraminidase, with 100 units of activity/ml. incubated for 24 hours at 37° C., were subjected to all treatments mentioned in the method (Sial. AB/PAS) but without any further effect.

*Normal tissues*

The superficial cells of the stratified squamous epithelium of the oesophagus contain glycogen granules which lose PAS reactivity completely after diastase digestion while the luminal surface is covered by a layer of sialomucin. The mucous secretion of the oesophageal glands is mainly formed of a neutral mucosubstance, a sialomucin and relatively smaller amounts of a sulphomucin.

In the stomach neutral mucosubstance is found in the surface epithelium, foveolar cells, cardiac and antral glands. Sialomucin is present in a few cells of the surface epithelium and foveolae as well as in most of the mucous neck cells. A sulphomucin is occasionally demonstrated in deep foveolar and mucous neck cells. Mucous neck cells are not always confined to sub-foveolar situation but occasionally extend to be wedged in between the cells of the fundic glands.

Goblet cells in all parts of the small intestine are found to secrete only an acid non-sulphated mucosubstance which is not fully labile to sialidase. Paneth cell granules reacted like neutral mucosubstances and all methods did not show any acid content.

Goblet cells along the surface of the colon and rectum contain mainly acid non-sulphated mucosubstances. More sulphomucin is seen in goblets of the luminal two-thirds of the caecum and appendix crypts than the deeper cells. The amount and distribution of sulphomucin vary greatly in the different segments of the colon and rectum. Ascending colon goblets show minimal amounts of sulphomucins. In other parts, for example the sigmoid colon, sulphomucins predominate in the deeper goblets of the crypts while an acid non-sulphated mucosubstance, not fully labile to sialidase, predominate in goblets in the upper parts of crypts. Goblet cells of the rectum, compared with those of the colon, show less sulphated material which is, in general, localised in the deep parts of the crypts.

The striated border reacts in a way which indicates the presence of a sialomucin only in the small intestine whereas in the colon and rectum, specially in the former, more sulphomucin is demonstrated. All luminal mucosubstance is acid non-sulphated in the small intestine, a mixture of both acid mucosubstances in the colon, and predominantly non-sulphated in the rectum.

**TABLE II.—*Histochemical Reactions of Normal Oesophageal Glands, Stomach, Small Intestine, Colon and Rectum***

| Tissue | Azure A pH 1·5 | Azure A pH 3·0 | PAS | Ph. Hyd. PAS | AB/PAS pH 2·5 | AB/PAS pH 1·0 | Meth. AB/PAS 37° C. | Meth. AB/PAS 60° C. | HID/AB | AF/AB | Sial. AB/PAS | Hyal. AB/PAS |
|---|---|---|---|---|---|---|---|---|---|---|---|---|
| Oesophageal glands | — | ++P | ++M | ++R | ++BP<br>+M | ++P<br>+M | ++BP<br>+M | ++B<br>+M | ++B<br>+Br | ++B<br>+P | ++RP<br>+B | ++BP<br>+M |
| Stomach | | | | | | | | | | | | |
| Gastric surface and superficial foveolar cells | — | — | +++M | — | +++M | ++M | ++M | ++M | ±B | ±B | ++R | +++M |
| Deep foveolar and mucous neck cells | — | — | +++M | ±R | ++M<br>+RP | ++M<br>±P | ++M<br>+P | ++M<br>±P | +B<br>±G | +B<br>±P | ++R<br>±B | ++M<br>+RP |
| Small intestine | | | | | | | | | | | | |
| Small intestinal goblet cells | — | +V | +++M | ±R | ++BP<br>+M | ++M<br>+P | ++BP<br>+M | ++MP<br>±B | ++B | ++B | ++RM<br>+B | ++BP<br>+M |
| Brunner's glands | — | — | ++M | — | ++M | ++RM | ++RM | ++RM | — | — | ++RM | ++M |
| Colon | | | | | | | | | | | | |
| Colonic goblet cells | — | ++V | +M | ±R | +++B | ++B<br>+M | +++B | ++B | ++B<br>++Br | ++B<br>++P | +++B | +++B |
| Rectum | | | | | | | | | | | | |
| Rectal goblet cells | — | ++P | +M | ±R | +++B | ++B<br>+RP | +++B | ++B | +++B<br>+Br | +++B<br>+P | ++B | +++B |

For abbreviations used in column headings see Methods section.

For significance of abbreviations in table see Results section.

**TABLE III.—*Histochemical Reactions of Human Oesophageal, Gastric, Colonic and Rectal Carcinomas***

| Tissue | Azure A pH 1·5 | Azure A pH 3·0 | PAS | Ph. Hyd. PAS | AB/PAS pH 2·5 | AB/PAS pH 1·0 | Meth. AB/PAS 37° C. | Meth. AB/PAS 60° C. | HID/AB | AF/AB | Sial. AB/PAS | Hyal. AB/PAS |
|---|---|---|---|---|---|---|---|---|---|---|---|---|
| Cancer oesophagus | — | — | ++M | +R | ++BP | +B<br>++P | +BP | +BP | ++B<br>+Br | ++B<br>+P | ++M<br>+B | ++BP |
| Cancer stomach | | | | | | | | | | | | |
| Moderately differentiated and undifferentiated carcinomas | — | ±V | +++M | ±R | +++B<br>++P | ++BP<br>+M | ++B<br>++P | ++BP<br>+M | +++B<br>+Br | +++B<br>+P | +++RM<br>+B | +++B<br>++P |
| Very well differentiated carcinomas | — | ++V | +++M | +R | +++B<br>+P | ++BM | ++B<br>+P | ++BP | ++Br<br>+B | +++P<br>+B | ++B<br>++M | +++P |
| Cancer colon | | | | | | | | | | | | |
| Moderately differentiated and non differentiated carcinomas | ±V | +++V | ++M | ±R | +++B<br>++P | ++BM | +++B<br>++P | ++B<br>++RP | +++B<br>+Br | +++B<br>+P | +++RM<br>+B | +++B<br>++P |
| Very well differentiated carcinomas | ±V | +++V | ++M | ±R | +++B<br>+P | ++BM | +++B<br>+P | ++B<br>+P | ++Br<br>+B | +++P<br>+B | ++B<br>+RM | +++B<br>+P |
| Cancer rectum | — | +++V | ++M | ±R | +++B<br>+P | ++BM | +++B<br>+P | ++B<br>+P | ++B<br>+Br | ++B<br>+P | ++B<br>+RM | +++B<br>+P |

For abbreviations used in column headings see Methods section.

For significance of abbreviations in table see Results section.

*Non-malignant polyps*

The hyperplastic cells of relatively small polyps of the stomach, colon or rectum contain variable amounts of an acid non-sulphated mucosubstance. Furthermore, the columnar epithelium of gastric and rectal polyps show a neutral mucosubstance in the supranuclear cytoplasm, while rectal and colonic polyps secrete a minimal amount of sulphomucin.

Large polyps in any of the three sites mentioned, produce a relatively larger amount of a sulphomucin in the cells and in cyst-like formations.

*Carcinoma*

The two cases of oesophageal carcinoma were both mucin-secreting adenocarcinomas of the lower third. Both acid non-sulphated mucosubstance and sulphomucin are demonstrated in tumour cells and secretion. Oesophageal glands in one of them show a large amount of sulphomucin.

The histological pattern of carcinoma of the stomach may vary greatly in the different parts of the same tumour, being differentiated in one area and undifferentiated in other areas. Mucin secretion is found to be common in both types. Mucosubstance is found either intracellularly, sometimes in signet-ring cells, or extracellularly in large accumulations surrounding groups of tumour cells or in cystic tumour acini.

In moderately differentiated and undifferentiated carcinomas malignant cells and secretion contain a sialomucin which is sialidase labile, whereas in well differentiated carcinomas variable amounts of a sulphomucin are identified. It is noted that the diffuse spheroidal-celled carcinomas, which are not well differentiated, either do not secrete or produce a secretion which is mainly a sialomucin, although in a few cases a sulphomucin is also present. On the other hand, in some very well differentiated growths the sialomucin content is either much greater than the sulphomucin or there is no sulphomucin at all.

Gastritic changes are found in the mucosa close to tumours and the incidence and extent of intestinal metaplasia is frequent and widespread. In several cases the cytoplasmic mucosubstance droplets in the epithelium bordering carcinomas and in intestinalised areas show increased sulphomucin and acid non-sulphated mucosubstance.

Foci of carcinoma *in situ* are found to be accompanied by areas of intestinalisation and to exhibit increased amounts of mucosubstances similar to those in well developed cancers, mainly sialomucin.

The structure of secondary deposits in lymph nodes and their reaction to the various histochemical techniques are more or less identical to the main type of the primary tumour.

Carcinomas of the colon and rectum gave comparable results. The characteristic of mucosubstances in these carcinomas depends on the degree of differentiation of the tumour. The higher the degree of differentiation in carcinoma of the colon and rectum the more closely mucosubstances resemble the normal.

In some carcinomas of the colon and rectum columnar cells are found to secrete a neutral mucosubstance at their supranuclear cytoplasm. The surrounding hyperplastic goblet cells in the colon and rectum contain a large amount of an acid non-sulphated mucosubstance and, only occasionally, a minimal amount of a sulphomucin.

*Connective tissue*

The connective tissue in polyps and in carcinoma shows a great increase in acid mucosubstance. This mucosubstance is, in most cases, completely digested by hyaluronidase. In some carcinomas it is not completely digested by sialidase or hyaluronidase but by consecutive treatment with both enzymes. The connective tissue within the nerve sheaths under the same conditions reacts in the same manner. The larger blood vessels show a subintimal and an adventitial deposition of a similar mucosubstance.

Many mast cells are found in tumours, chiefly in the connective tissue and underlying muscle but occasionally infiltrate masses of tumour cells. Their staining reactions indicate the presence of a sulphomucin.

### DISCUSSION

*Normal tissues*

The layer of sialomucin which has been found to cover the luminal surface of the oesophagus is probably derived from the oesophageal glands since the oesophageal epithelium has been shown to be devoid of any mucosubstances.

The surface epithelium of the stomach together with the foveolar cells, cardiac and gastric glands are the source of the bulk of neutral mucosubstance secreted by the stomach. The main origin of sialomucin is the mucous neck cells although the foveolar cells and surface epithelium contribute to it. The deep foveolar and mucous neck cells elaborate the rather small amount of sulphomucin secreted by the normal gastric mucosa.

The extension of the mucous neck cells between the cells of the fundic glands has been observed by Lev (1966) who has come to the same conclusions as stated above except that he did not point to the presence of sialomucins in the surface epithelium.

Acid mucosubstances in mucous neck cells and other epithelial cells of the stomach have been demonstrated by mucicarmine (Bensley, 1898) and colloidal iron and alcian blue (Mowry and Jones, 1959; Belanger, 1963; Lillibridge, 1964).

Gastric mucosubstances have been the subject of extensive chemical investigation, and were found to be rich sources of blood group substances (Meyer *et al.*, 1937; Morgan and King, 1943; Glass, 1949; Komarov, 1952; Hollander, 1962; Kent, 1962). Glass (1962) identified sulphate, sialic and uronic acids in mucus obtained from healthy and diseased human stomachs. Other authors have confirmed the presence of sulphomucins (Meyer *et al.*, 1937; Werner, 1953; Komarov, 1952; Kent and Whitehouse, 1955; Kent, 1962) and sialomucins (Werner, 1953; Hollander, 1962; Kent, 1962; Kent, 1963; Glass, 1963; Hoskins and Zamcheck, 1963) in gastric mucosa. Human gastric sulphomucin has been found by Schrager (1964) to be associated with hexosamine. Meyer *et al.* (1937) and Werner (1953) considered uronic acid as one of the components of sulphomucins of gastric secretion, but Gottschalk (1960) and Schrager (1963) could not confirm this. Furthermore, uronic acid has not been demonstrated in any epithelial mucosubstance except keratosulphate (Dorfman, 1963). The comparatively large amounts of sulphate and uronic acid which have been reported in chemical investigations of gastric mucosa are most likely to be derived from the connective tissue as much of the chemical work has been done on extracts of mucosa which might contain mesenchymal mucosubstances.

Gottschalk *et al.* (1965) have confirmed by autoradiography of human tissue the existence of sulphate in gastric mucosa and secretion.

The finding that goblet cells of the small intestine secrete an acid non-sulphated mucosubstance only, agrees with the result obtained by Lev and Spicer (1965).

Human Paneth cell granules have been found to contain only a neutral mucosubstance. Similarly, rat Paneth cell granules have been shown to be devoid of acid mucosubstances (Taylor and Flaa, 1964) but to react with PAS (Leblond, 1950).

Results obtained by Lev and Spicer (1965) about the distribution of both types of acid mucosubstances in the different parts of the colon and rectum are similar to results recorded in this work. Even with the empirical methods, Lauren (1961) has been able to demonstrate staining differences in the goblet cells of the small intestine.

Moog and Wenger (1952) advanced the idea that the rods of the striated border are composed mainly of a network of neutral mucosubstances and did not recognise any acid mucosubstances in them. Luke and Spicer (1965) have stated that the human gastro-intestinal epithelium is coated with a surface layer comprising both sialo- and sulphomucins.

The chemical composition of mucosubstances of the human intestinal tract is not fully revealed. Hoskins and Zamcheck (1963) have identified sialic acid in duodenal and rectal mucosubstances. Werner (1953) considered neutral mucosubstance as the main constituent of pig intestinal mucosubstances and a sulphomucin, probably containing mucoitin sulphuric acid, and a sialomucin as subsidiary components. The latter two materials have been found in sheep colonic mucosubstance but the presence of mucoitin sulphate is questioned (Kent, 1963).

Human intestinal mucosa and secreted mucosubstance have been demonstrated by Gottschalk *et al.* (1965) to incorporate more radioactive sulphate than gastric mucosa and secretion.

*Non-malignant polyps*

Small gastric polyps have been said (Lev, 1966) to produce less mucosubstance than normal epithelium and acidic mucosubstances have not been mentioned to be present in both types of polyps, small or large. Gottschalk *et al.* (1965) have demonstrated autoradiographically the uptake of sulphate by a colonic polyp. It has been suggested that malignant changes are only likely to develop in large polyps (Hay, 1953; Stout, 1953; Monaco *et al.*, 1962; Ming and Goldman, 1965; Lev, 1966). The increase of sulphomucins reported in large polyps in the present paper lends histochemical support to this theory, since sulphomucins have been found to be present in increased amounts in many carcinomas.

*Carcinoma*

The great variation in the histological pattern of carcinoma of the stomach in different parts of the same tumour has been observed by Stout (1943, 1953), Muir (1958), Ackerman and Del Regato (1962) and Lauren (1965). Moreover, secretion and distribution of mucosubstances in the different tumour types is confirmed by Muir (1958), Robbins (1962) and Lauren (1965).

The increased amount of sulphomucins in carcinomatous gastric tissue and its secretion is, generally speaking, in accord with the result reported by Lev (1966). Nevertheless, he did not discuss the existence and relative variation of this type of mucosubstance according to the degree of differentiation or de-differentiation of the tumour. Sialomucin has been found to be more prevalent in moderately differentiated and undifferentiated carcinomas, whereas sulphomucin dominated the picture in well differentiated growths.

It has been noted in previous studies of different organs that secretion of mucosubstance by a tumour is inversely proportional to the grade of malignancy (Ochsenhirt, 1928; Lauren, 1961; Garcia-Bunuel and Monis, 1964; Hukill and Vidone, 1965). This has been observed in tissues studied and the undifferentiated tumours have been found either not to secrete or to produce a secretion which is mainly a sialomucin although in few cases a sulphomucin, as well, has been shown.

The acid non-sulphated mucosubstance in malignant tissues, contrary to what has been found by Lev (1966) proved to be sialidase-susceptible indicating that it is definitely a sialomucin.

The presence of acid mucosubstances in foci of carcinoma *in situ* has not been mentioned in the available literature, whereas the existence of acid mucosubstances in gastric carcinoma have been reported in previous work using old histochemical techniques by Mowry and Jones (1959), Lauren (1965) and Dobrogorski and Braunstein (1963) who did not succeed in differentiating the two components of this type of mucosubstance, the sialo- and sulphomucin. The increase of sialic acid in carcinomatous tissue has been shown chemically by Richmond *et al.* (1955) and Barker *et al.* (1959) and the presence of sulphomucin by autoradiographic investigation of human tissue by Gottschalk *et al.* (1965).

The increased sulphomucin in epithelium bordering carcinoma confirms the finding by Lev (1966). Intestinalisation of gastric mucosa close to polyps and tumours have been reported by Guiss and Stewart (1943), Stout (1953), McManus and Mowry (1960), Jarvi and Lauren (1951), Morson (1955*a*), Lauren (1961, 1965), Cruickshank *et al.* (1964) and Lev (1966). It has been maintained by Lev and Spicer (1965) and Lev (1966) that intestinalised epithelial areas are structurally and histochemically similar to the epithelium of the small intestine. Stout (1953) differentiated between mucosubstances secreted by intestinalised areas and gastric mucosubstance as the former stained with mucicarmine while the latter did not. Basophilia of intestinalised epithelium has been found by Lev and Spicer (1965) to be more labile to sialidase than that of small intestinal goblet cells which is exactly opposite to findings in this work. Sulphomucin has been demonstrated in goblet cells of intestinalised gastric epithelium. Many authors (Jarvi and Lauren, 1951, 1952; Morson, 1955*b*, 1962; Mulligan and Rember, 1954; Lev 1966) have advanced the idea that there is a direct relationship between the presence of intestinalised epithelium and the incidence of gastric carcinoma. However, Mowry and Jones (1959) and Planteydt and Willighagen (1965) denied any direct relation between metaplasia and gastric carcinoma. The former believed that carcinoma originates from the proliferative portions of normal gastric mucosa, whereas the latter suggested that it is due to faulty regeneration in gastric mucosa which is the same cause as for intestinal metaplasia. Stout (1953) stated that intestinalisation is a frequent gastric change in advanced age.

The presence of sialomucins and sulphomucins in carcinomas of the oesophagus, colon and rectum simulates the distribution of these mucosubstances in carcinoma of the stomach. Furthermore, the occurrence of large amounts of sialidase resistant acid mucosubstance in the surrounding hyperplastic goblet cells in the colon and rectum has been noted in gastric carcinoma.

Ochsenhirt (1928) has been able to demonstrate mucosubstances stained with mucicarmine in carcinomatous tissue of the large intestine. Sulphate uptake has been demonstrated in human carcinomas of the colon by Gottschalk *et al.* (1965).

The constant existence of acidic mucosubstances, particularly sulphomucins, in carcinomatous tissues of the alimentary tract irrespective of the type of mucosubstance secreted by the organ characterises this type of lesion. This probably represents a disturbance in the normal secretion cycle rather than a direct effect of the malignant process itself. The presence of sialomucin in malignant tissue has been verified histochemically in this work confirming chemical studies and disagreeing with the suggestion that sialomucin is increased due to secretion from intestinalised epithelium (Lev, 1966). Mucosubstance from intestinalised areas has been found to be sialidase resistant.

The establishment of these characteristics of behaviour of the alimentary tract mucosa in the tumours studied may be of diagnostic significance.

The conclusion that the structure of secondary deposits in lymph nodes and their mucosubstances are similar to the main type of the primary tumour has been made before by Franks *et al.* (1964) and Lauren (1965). Gottschalk *et al.* (1965) observed by autoradiography that the sulphate concentration in metastases varied moderately from that in the primary growth. Cutaneous metastases of a cancer colon have proved to contain mucosubstances similar to the primary growth (Johnson and Helwig, 1963*a*, *b*). An attempt to recognise the primary tumour by means of characterising the mucosubstance of the secondaries has been made by Foster and Levine (1963). This is perhaps easier to apply after the application of the new histochemical techniques which differentiate between the different types of acid mucosubstances.

*Connective tissue*

The increased amounts of acidic mucosubstances in the connective tissue stroma of polyps, and in carcinoma, as well as in nerve sheaths and larger blood vessels in such conditions particularly in carcinoma have been noted by Hukill and Vidone (1965) and Johnson and Helwig (1963*a*). Bunting and White (1950) stated that the increase of hyaluronic acid should be expected in rapidly growing connective tissue which is the case in the conditions mentioned above. Sulphate has been shown autoradiographically in the stroma of human intestinal carcinoma (Gottschalk *et al.*, 1965).

Results in this work point to the presence of sialic acid in addition to hyaluronidase-susceptible mucosubstance in the stroma of some carcinomas. This may be elaborated by the glandular elements which had invaded the connective tissue.

Findings about the presence of abundant numbers of mast cells and their reaction to different stains and techniques as well as their distribution in malignant tissue confirm those reported by Hukill and Vidone (1965) in carcinoma of the bladder.

SUMMARY

The histochemical characteristics of the mucosubstances of the normal tissue, benign polyps and carcinomas of the human alimentary tract have been investigated using both empirical and modern histochemical techniques.

Normal oesophageal epithelium has been found not to secrete any mucosubstance. The neutral mucosubstance of the stomach is elaborated by the surface epithelium, foveolar cells, cardiac and gastric glands. Mucous neck cells secrete both sialo- and sulphomucins. Foveolar cells and surface epithelium contribute to the amount of sialomucin formed by the stomach while the deep foveolar cells secrete a small amount of sulphomucin.

Small intestinal goblet cells contain an acid non-sulphated mucosubstance which is partially resistant to sialidase. Colonic and rectal goblet cells secrete an acid non-sulphated sialidase resistant mucosubstance and a sulphomucin. Sulphomucins are more prevalent in the colon than in the rectum.

It has been found that sulphomucins increase in larger polyps and well differentiated carcinomatous tissue, whereas sialomucins prevail in moderately differentiated and undifferentiated carcinomas. The acid non-sulphated mucosubstance in malignant tissue has been found to be a sialomucin. Mucosubstance in the secondary deposits in lymph nodes has been shown to be similar to the main type of mucosubstance in the primary tumour. It is suggested that these findings may be of help in diagnosing carcinomas of the alimentary tract.

### REFERENCES

Ackerman, L. V. and Del Regato, J. A.—(1962) " Cancer. Diagnosis, treatment and prognosis ". Saint Louis (The C. V. Mosby Company).

Barker, S. A., Stacey, M., Tipper, D. J. and Kirkham, J. H.—(1959) *Nature, Lond.*, **184**, 68.

Belanger, L. F.—(1963) *Ann. N.Y. Acad. Sci.*, **106**, 364.

Bensley, R. R.—(1898) *Q. Jl microsc. Sci.*, **41**, 361.

Bunting, H. and White, R. F.—(1950) *A.M.A. Archs Path.*, **49**, 590.

Cruickshank, B., Dodds, T. C. and Gardner, D. L.—(1964) " Human histology ". Edinburgh and London (E. and S. Livingstone Ltd).

Dobrogorski, O. J. and Braunstein, H.—(1963) *Am. J. clin. Path.*, **40**, 435.

Dorfman, A.—(1963) *J. Histochem. Cytochem.*, **11**, 2.

Foster, E. A. and Levine, A. J.—(1963) *Cancer, N.Y.*, **16**, 506.

Franks, L. M., O'Shea, J. D. and Thomson, A. E. R.—(1964) *Cancer, N.Y.*, **17**, 983.

Garcia-Bunuel, R. and Monis, B.—(1964) *Cancer, N.Y.*, **17**, 1108.

Glass, G. B. J.—(1949) *Rev. Gastroent.*, **16**, 687.—(1962) *Gastroenterology*, **43**, 310.—(1963) *Ann. N.Y. Acad. Sci.*, **106**, 775.

Gottschalk, A.—(1960) " The chemistry and biology of sialic acids and related substances ". London and New York (Cambridge Univ. Press).

Gottschalk, R. G., Bell, P. and Miller, P. O.—(1965) *Cancer Res.*, **25**, 911.

Guiss, L. W. and Stewart, F. W.—(1943) *Archs Surg.*, **46**, 823.

Hay, L. J.—(1953) *Surgery, St. Louis*, **33**, 446.

Hollander, F.—(1962) *Gastroenterology*, **43**, 304.

Hoskins, L. C. and Zamcheck, N.—(1963) *Ann. N.Y. Acad. Sci.*, **106**, 767.

Hotchkiss, R. D.—(1948) *Archs Biochem.*, **16**, 131.

Hukill, P. B. and Vidone, R. A.—(1965) *Lab. Invest.*, **14**, 1624.

Jarvi, O. and Lauren, P.—(1951) *Acta path. microbiol. scand.* (Suppl.), **29**, 26.—(1952) *Acta Un. Int. Cancr.*, **8**, 393.

Jennings, M. A. and Florey, H. W.—(1956) *Q. Jl exp. Physiol.*, **41**, 131.

Johnson, W. C. and Helwig, E. B.—(1963*a*) *Am. J. clin. Path.*, **40**, 123.—(1963*b*) *Ann. N.Y. Acad. Sci.*, **106**, 794.

Kent, P. W.—(1962) *Gastroenterology*, **43**, 292.

KENT, P. W. AND WHITEHOUSE, M. W.—(1955) "Biochemistry of the amino-sugars". London (Butterworths Scientific Publications).
KENT, S. P.—(1963) *J. Histochem. Cytochem.*, **2**, 273.
KOMAROV, S. A.—(1952) "Mucoproteins of gastric secretions". In: Proceedings of Second National Cancer Conference. New York (American Cancer Society Inc.), **1**, 799.
LAUREN, P.—(1961) *Acta path. microbiol. scand.* (Suppl.), **152**, 9.—(1965) *Acta path. microbiol. scand.* (Suppl.), **64**, 31.
LEBLOND, C. P.—(1950) *Am. J. Anat.*, **86**, 1.
LEV, R.—(1966) *Lab. Invest.*, **14**, 2080.
LEV, R. AND SPICER, S. S.—(1965) *Am. J. Path.*, **46**, 23.
LILLIBRIDGE, C. B.—(1964) *Gastroenterology*, **47**, 269.
LILLIE, R. D.—(1954) "Histopathologic technic and practical histochemistry". 2nd Edition. New York (The Blakiston Co.).—(1964) *J. Histochem. Cytochem.*, **12**, 821.
LUKE, J. L. AND SPICER, S. S.—(1965) *Lab. Invest.*, **14**, 2101.
MCMANUS, J. F. A. AND MOWRY, R. W.—(1960) "Staining methods. Histological and histochemical". New York (Paul B. Hoeber, Inc.).
MEYER, K., SMYTH, E. M. AND PALMER, J. W.—(1937) *J. biol. Chem.*, **119**, 73.
MING, S. C. AND GOLDMAN, H.—(1965) *Cancer, N.Y.*, **18**, 721.
MONACO, A. P., ROTH, S. I., CASTLEMAN, B. AND WELCH, C. E.—(1962) *Cancer N.Y.*, **15**, 456.
MOOG, F. AND WENGER, E. L.—(1952) *Am. J. Anat.*, **90**, 339.
MORGAN, W. T. J. AND KING, H. K.—(1943) *Biochem. J.*, **37**, 640.
MORSON, B. C.—(1955*a*) *Br. J. Cancer*, **9**, 365.—(1955*b*) *Br. J. Cancer*, **9**, 377.—(1962) *J. Am. med. Ass.*, **179**, 311.
MOWRY, R. W. AND JONES, P. B.—(1959) *J. Histochem. Cytochem.*, **7**, 321.
MOWRY, R. W. AND WINKLER, C. H.—(1956) *Am. J. Path.*, **32**, 628.
MUIR, R.—(1958) "Alimentary system". In: Muir's textbook of pathology, 7th Edition. London (Edward Arnold (Publishers) Ltd).
MULLIGAN, R. M. AND REMBER, R. R.—(1954) *A.M.A. Archs Path.*, **58**, 1.
OCHSENHIRT, N. C.—(1928) *Surgery Gynec. Obstet.*, **47**, 32.
PLANTEYDT, H. T. AND WILLIGHAGEN, R. G. J.—(1965) *J. Path. Bact.*, **90**, 393.
QUINTARELLI, G.—(1963) *Ann. N.Y. Acad. Sci.*, **106**, 339.
QUINTARELLI G., TSUIKI, S., HASHIMOTO, Y. AND PIGMAN, W. (1961) *J. Histochem. Cytochm.*, **9**, 176.
RICHMOND, V., CAPUTTO, R. AND WOLF, S.—(1955) *Gastroenterology*, **29**, 1017.
ROBBINS, S. L.—(1962) "Text book of pathology with clinical application", 2nd Edition. Philadelphia and London (W. B. Saunders Company).
SCHRAGER, J.—(1963) *Nature, Lond.*, **198**, 899.—(1964) *Nature, Lond.*, **201**, 702.
SPICER, S. S.—(1960) *J. Histochem. Cytochem.*, **8**, 18.—(1961) *Am. J. clin. Path.*, **36**, 393.—(1962) *Annls Histochim.*, **7**, 23.—(1965) *J. Histochem. Cytochem.*, **13**, 211.
SPICER, S. S. AND DUVENCI, J.—(1964) *Anat. Rec.*, **149**, 333.
SPICER, S. S. AND JARRELS, M. H.—(1961) *J. Histochem. Cytochem.*, **9**, 368.
SPICER, S. S. AND MEYER, D. B.—(1960) *Am. J. clin. Path.*, **33**, 453.
SPICER, S. S. NEUBECKER, R. D., WARREN, L. AND HENSON, J. G.—(1962) *J. natn. Cancer Inst.*, **29**, 963.
STOUT, A. P.—(1943) *Archs Surg., Chicago*, **46**, 807.—(1953) "Tumours of the stomach". In: "Atlas of tumour pathology", Section 6, Fascicle 21. Washington, D.C. (Subcommittee on Oncology of the Committee on Pathology of the National Research Council, Armed Forces Institute of Pathology).
TAYLOR, J. J. AND FLAA, R. C.—(1964) *A.M.A. Archs Path.*, **77**, 278.
TOCK, E. P. C. AND PEARSE, A. G. E.—(1965) *Jl R. microsc. Soc.*, **84**, 519.
WERNER, I.—(1953) *Acta Soc. Med. upsal.*, **58**, 1.